# Fabrication and Characterization of Acute Myocardial Infarction Myoglobin Biomarker Based on Chromium-Doped Zinc Oxide Nanoparticles

**DOI:** 10.3390/bios12080585

**Published:** 2022-08-01

**Authors:** Adel Al Fatease, Mazharul Haque, Ahmad Umar, Shafeeque G. Ansari, Mater H. Mahnashi, Yahya Alhamhoom, Zubaida A. Ansari

**Affiliations:** 1 Department of Pharmaceutics, College of Pharmacy, King Khalid University, Guraiger, Abha 62529, Saudi Arabia; afatease@kku.edu.sa (A.A.F.); ysalhamhoom@kku.edu.sa (Y.A.); 2 Centre for Interdisciplinary Research in Basic Sciences, Jamia Millia Islamia, Jamia Nagar, New Delhi 110025, India; mhaque3@jmi.ac.in (M.H.); saansari@jmi.ac.in (S.G.A.); zaansari@jmi.ac.in (Z.A.A.); 3 Department of Chemistry, Faculty of Science and Arts and Promising Centre for Sensors and Electronic Devices (PCSED), Najran University, Najran 11001, Saudi Arabia; 4 Department of Materials Science and Engineering, The Ohio State University, Columbus, OH 43210, USA; 5 Department of Pharmaceutical Chemistry, College of Pharmacy, Najran University, Najran 61441, Saudi Arabia; mhmahneshi@nu.edu.sa

**Keywords:** acute myocardial infarction, myoglobin biosensor, biomarker, chromium-doped ZnO

## Abstract

In this article, we describe the fabrication and characterization of a sensor for acute myocardial infarction that detects myoglobin biomarkers using chromium (Cr)-doped zinc oxide (ZnO) nanoparticles (NPs). Pure and Cr-doped ZnO NPs (13 × 10^17^, 20 × 10^17^, and 32 × 10^17^ atoms/cm^3^ in the solid phase) were synthesized by a facile low-temperature sol-gel method. Synthesized NPs were examined for structure and morphological analysis using various techniques to confirm the successful formation of ZnO NPs. Zeta potential was measured in LB media at a negative value and increased with doping. XPS spectra confirmed the presence of oxygen deficiency in the synthesized material. To fabricate the sensor, synthesized NPs were screen-printed over a pre-fabricated gold-coated working electrode for electrochemical detection of myoglobin (Mb). Cr-doped ZnO NPs doped with 13 × 10^17^ Cr atomic/cm^3^ revealed the highest sensitivity of ~37.97 μA.cm^−2^nM^−1^ and limit of detection (LOD) of 0.15 nM for Mb with a response time of ≤10 ms. The interference study was carried out with cytochrome c (Cyt-c) due to its resemblance with Mb and human serum albumin (HSA) abundance in the blood and displayed distinct oxidation potential and current values for Mb. Cr-doped ZnO NP-based Mb biosensors showed 3 times higher sensitivity as compared to pure ZnO NP-based sensors.

## 1. Introduction

Cardiovascular diseases (CVDs) are caused by anomalies in the heart and blood vessels, leading to a variety of disorders associated with blood supply to the heart muscle through arteries causing damage to heart muscles, valves, and other body components that ultimately lead to heart stroke [1,2]. This recently put forward the demand for an early detection of cardiovascular disorders such as acute myocardial infarction (AMI) [3,4]. Since early detection saves lives as well as reduces socioeconomic burden worldwide as costly and time-consuming, diagnostic procedures are avoided [5]. In the last decade, globally, scientists have exploited techniques to develop low-cost solutions for the detection of various cardiac biomarkers [6,7]. Electrochemical sensors/transducers being low cost, fast and efficient have been extensively researched [8,9,10,11]. Moreover, nanomaterials-based sensors show the potential for economically viable solutions to cut down time consumption and costs of early detection and cures for cardiovascular diseases [12,13,14,15,16,17,18].

To detect cardiovascular disease, myoglobin (Mb) is one of the biomarkers as its levels rise in the serum beyond the healthy range of 70 (90) ng/mL (4/5 nM) to 200 ng/mL (11 nM) [19]. The increase in Mb content by 4–10 times in the bloodstream signifies acute myocardial damage. The rise of Mb levels from baseline occurs within 3 h from the advent of initial symptoms of acute myocardial infarction (AMI) and achieves the maximum value in the time span of 6–12 h. The detection of Mb levels is important to analyze AMI at an early stage to prevent the severity and development of heart attacks or other cardiovascular illnesses [19,20]. Therefore, Mb is considered one of the earliest markers for AMI detection and confirmation [20].

To develop a rapid and highly sensitive detection system for Mb, nanomaterials can be used as they exhibit potential for such devices. Nanomaterials have been employed as a rapid charge-transfer matrix during the fabrication of highly sensitive biosensors owing to their large surface-to-volume ratios, which provide a large density of binding sites to biomolecules resulting in improved sensing performance [12,13]. Size compatibility of nanomaterials with biomolecules leads to better immobilization over the surface of nanostructured materials [14,15,16]. Nanomaterials-based biosensors are intriguing due to their potential for detecting sub-nanomolar concentrations using a few microliters of volume [17,18]. Nanomaterials-based biosensors are illustrated as potential replacements for conventional devices with high sensitivity, rapid response, and low detection limit [6,21,22].

High bandgap nanomaterials are preferably used for sensing applications due to their excellent charge-transfer characteristics resulting in good quality electrical signals [1]. Zinc oxide (ZnO) with a bandgap of over 3.3 eV and binding energy of exciton of 60 meV is a promising nanostructured semiconductor metal-oxide used for various applications such as energy, sensor, electronic, optoelectronics, medicinal, and biological devices including biosensors [1,23,24,25,26,27,28]. The bandgap of ZnO has been tailored and the oxygen concentration is varied by doping with different metal ions such as tin (Sn), cobalt (Co), copper (Cu), nickel (Ni), manganese (Mn), magnesium (Mg), etc., to improve various physical properties [29,30,31,32,33,34,35,36]. Doping, therefore, provides a practical way to tune physical properties, particularly optical, electrical, magnetic, charge transport, and electrochemical properties to meet specific requirements [37,38,39,40,41,42]. Besides this, doping also influences the crystal size, surface potential, semiconductor energy levels, and carrier concentration that are utilized for sensors [9,43]. Interestingly, doping was found to improve the characteristics of doped ZnO when compared with pure material.

Because of its close ionic radius proximity to Zn^2+^ ions, doping of Cr^3+^ metal ions into ZnO has recently received a lot of interest [44,45,46,47,48]. The ionic radius of Cr^3+^ (0.062 nm) and Zn^2+^ (0.072 nm) is practically identical; thus, Cr^3+^ ions can seamlessly replace Zn^2+^ ions in the lattices of ZnO-inducing strain and hence defects in the crystal of the parent material preserving the structure [44]. Interestingly, Cr^3+^ also introduces a significant number of electrons into the doped ZnO because it contains multiple electron shells and thus, increases the performance of the devices [44,45,46]. Because of their high-performance characteristics, Cr-doped ZnO nanomaterials are used for a variety of applications and reported in the literature [44,45,46,47,48]. Using Cr-doped ZnO nanoparticles, Nakarungsee et al. fabricated a highly sensitive ammonia gas sensor operated at room temperature [44]. Iqbal et al. used hierarchical Cr-doped ZnO nanostructures for photovoltaic application which exhibited a light-to-electricity conversion efficiency of ~0.79% [45]. As a photocatalyst, Cr-doped ZnO nanorods arrays exhibited a complete photocatalytic degradation performance towards highly toxic organic dye Direct blue 86 (DB86) [46]. A high-performance acetone sensor was fabricated using Cr-doped ZnO nanofilms by Al-Hardan et al. and reported in the literature [47]. Chinnasamy et al. demonstrated the use of Cr-doped ZnO nanorods for enhanced UV photodetection [48]. Although used for a variety of applications, Cr-doped ZnO nanomaterials are never used for biosensor applications, especially for Mb biomarkers.

Herein, we report the fabrication and characterization of a myoglobin biomarker sensor to detect acute myocardial infarction using chromium (Cr)-doped zinc oxide (ZnO) NPs. Synthesized Cr-doped ZnO NPs were characterized for their various properties prior to sensor fabrication. An electrochemical method was adopted for analyzing the performance of the fabricated myoglobin biosensor. To the best of our knowledge, the use of Cr-doped ZnO NPs to fabricate myoglobin biomarkers has never been reported in the literature. Mb detection of low concentration ensured in this study provides a strong case for further investigation of the biosensing potential associated with semiconductor oxides.

## 2. Experimental Details

### 2.1. Materials

Zinc acetate (Zn (CH_3_COO)_2_·2H_2_O; 98%), chromium nitrate (Cr(NO_3_)_3_·9H_2_O; 98%), and NaOH were procured from Loba chemicals, India. Myoglobin (100684-32-0) and HSA (70024-90-7) were procured from Sigma-Aldrich, St. Louis, MO, USA. To prepare phosphate buffer, (NaH_2_PO_4_·2H_2_O, 98%) and (Na_2_HPO_4_, 99%) were obtained from Fischer Scientific, India. Deionized (DI) water with a resistivity of 18.3 MΩ (Millipore) was used for preparing all the solutions.

### 2.2. Synthesis of ZnO and Cr-Doped ZnO NPs

ZnO NPs were synthesized using an altered sol-gel method [49]. In a typical reaction, 2 mM of Zn (CH_3_COO)_2_·2H_2_O was dissolved in 40 mL ethanol at room temperature and stirred for 20 min. Further, 40 mL of 4.0 M NaOH ethanol solution was added dropwise into the above solution under stirring. In the resulting solution, specific amounts of Cr(NO_3_)_3_·9H_2_O (0.08, 0.15, and 0.22 mM) were added as dopant precursors to incur final 13 × 10^17^, 20 × 10^17^, and 32 × 10^17^ Cr atoms/cm^3^, in three separate reactions. After proper stirring, the resultant solution was transferred into a three-neck flask for refluxing at 60 °C for 3 h. The final precipitate was formed which was decanted and washed out with DI water and subsequently with ethanol and dried at 70 °C for 6 h. The prepared powder was further characterized by different methods in order to elucidate its structural, optical, morphological, and other properties.

### 2.3. Characterizations

The surface morphologies of the synthesized ZnO powders were analyzed using field emission scanning electron microscopy (FESEM; SU 70, Hitachi, Japan). The structural analysis of as-synthesized NPs was performed using an X-ray diffractometer (XRD; Ultima IV, Rigaku) availing Cu-K*α* (*λ* = 1.542 Å) and the diffraction spectra were obtained at a Bragg’s angle between 20 and 80 degrees. Particle size ‘D’ was estimated using Debye Scherer’s formula [49]. The induced strain in the grown crystals led by a 5% lattice mismatch was estimated from Williamson–Hall (W–H) relation [49].

The optical properties of materials were examined using a UV–Vis (U3900, Hitachi) spectrophotometer in the wavelength range of 225–450 nm. Surface compositions were probed by Fourier transform infrared (FTIR; Tensor 37 spectrophotometer, Bruker, Billerica, MA, USA) at room temperature in ATR mode. X-ray photoelectron spectroscopy (XPS, AXIS-NIVA CJ 109, Kratos, Manchester, UK) was carried out to investigate the surface elemental and electronic structure of synthesized NPs. The zeta potential on the surface of NPs for all samples was measured separately using a Zeta Sizer Nano ZS (Malvern Instruments Ltd., Worcestershire, UK) using an optimized concentration of 2 mg/mL in LB media using a HeNe laser as the source.

### 2.4. Fabrication of Mb Sensor

To fabricate the electrochemical sensor for Mb, a slurry of ZnO NPs made with organic binders was coated on the prefabricated gold-plated electrodes. Nanoparticle paste was poised in the agate mortar and pestle using fine powder and drop-by-drop addition of organic binders, i.e., a 70:30 mixture of ethyl cellulose and butyl carbitol acetate. The coated electrode was kept for film settling for 30 min and dried for 4 h at 60 °C. The electrochemical activities of fabricated electrodes were investigated by cyclic voltammetry (CV; IVIUM’s Potentiostat) for potential ranging from −1.0 V to +1.0 V at many scan rates. For each concentration and all samples, the CV results were obtained in triplicate. Mb solutions of various concentrations were prepared in 0.1 M, 7.2 pH phosphate buffer solution and analyzed individually. The peak current plotted over Mb concentration was used as a calibration curve to calculate sensitivity and unknown concentrations. In addition, charge-transfer properties for all fabricated sensors were analyzed using electrochemical impedance spectroscopy (EIS). All the electrochemical experiments were performed at room temperature.

## 3. Results and Discussion

### 3.1. Characterizations and Properties of Cr-Doped ZnO NPs

**Figure 1:**
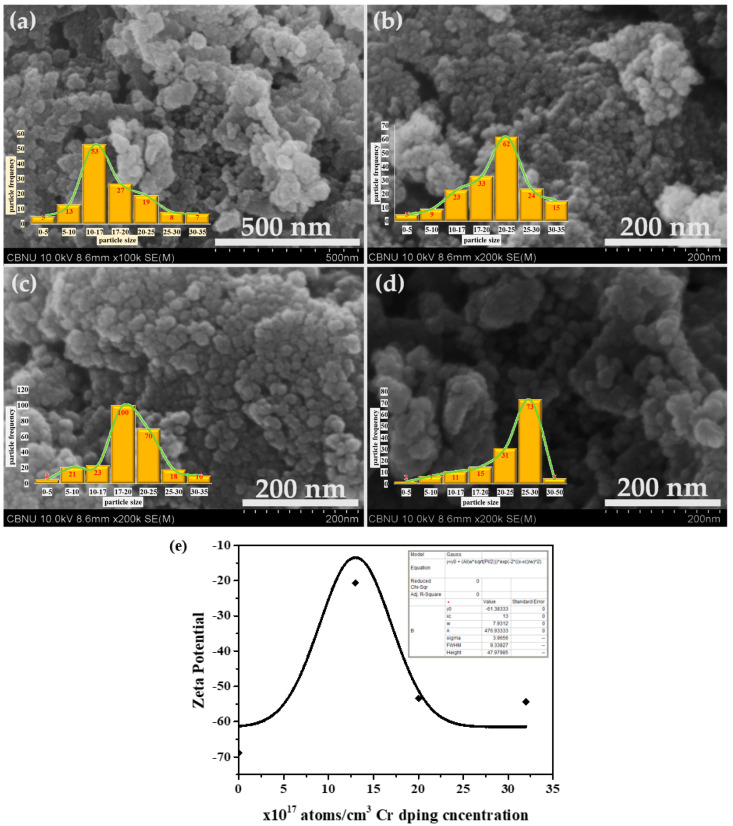
Typical FESEM images of synthesized pure ZnO and Cr-doped ZnO NPs. (**a**) Pure ZnO; (**b**) ZnO-Cr (Cr = 13 × 10^17^ atoms/cm^3^); (**c**) ZnO-Cr (Cr = 20 × 10^17^ atoms/cm^3^); and (**d**) ZnO-Cr (Cr = 32 × 10^17^ atoms/cm^3^). (**e**) Zeta potential graph for all synthesized nanoparticles.

The general morphologies of the synthesized materials were examined by field emission scanning electron microscopy (FESEM). Figure 1 depicts the typical FESEM micrographs of the synthesized pure ZnO and Cr-doped ZnO materials. The observed micrographs clearly confirmed the formation of spherical-shaped morphologies in nano-dimensions, thus, named “NPs”. The size distribution of the particles given in the micrographs shows uniformity. The sizes of the NPs are almost uniform, i.e., 25 ± 5 nm except for slight aggregation of the sample with the highest Cr doping concentration. Interestingly, chromium (Cr) doping does not affect the shape and size of the synthesized NPs. The zeta potential on the surface was measured using a zeta sizer (Marvel ZS) and found the lowest value of −68.8667 mV for pristine ZnO and the highest for Cr-ZnO with a minimum doping concentration which follows the trend as that of the density of states confirming the presence of a comparatively high density of free electrons on the surface amid the stress.

**Figure 2:**
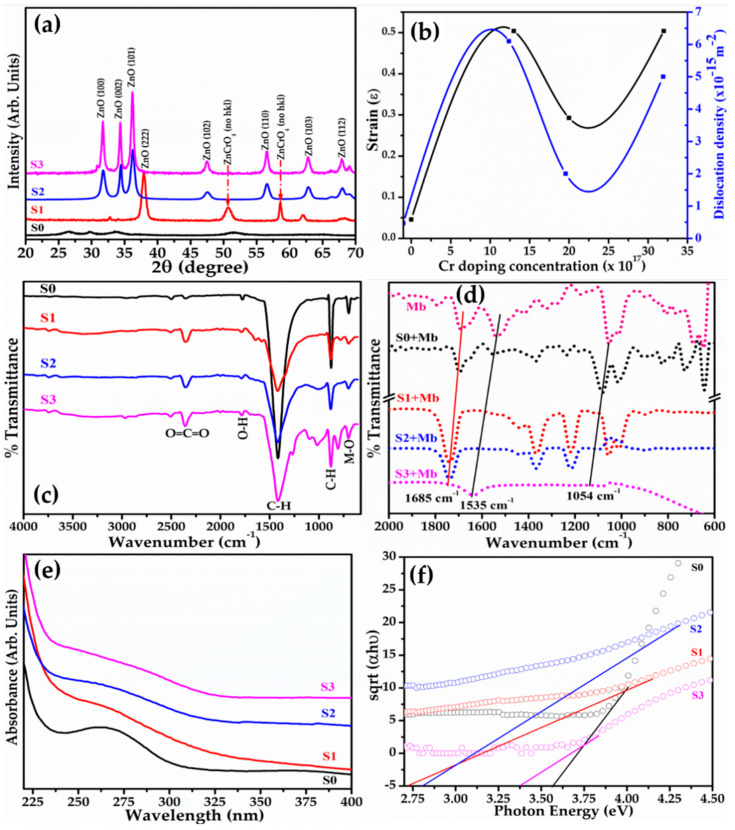
Typical (**a**) X-ray diffraction pattern; (**b**) strain and dislocation density; (**c**) Fourier transform-IR spectra; (**d**) FTIR spectra of synthesized NPs titrated with Mb; (**e**) set of UV–Vis absorption spectra; and (**f**) Tauc’s plot of synthesized pure ZnO and Cr-doped ZnO NPs.

The crystal properties and crystallinity of the prepared NPs were examined by X-ray diffraction analysis. Figure 2a depicts the diffraction pattern of the pure and Cr-doped ZnO NPs. Various well-defined diffraction peaks related to ZnO planes (100), (002), (101), (222), (102), (103), and (112) are observed in pure ZnO whereas in Cr-doped ZnO additional peaks related to unidentified planes of ZnCrO_4_ are observed that are confirmed from standard JCPDS card 89-7102 and 13-0311. The sharp intense peaks of the diffraction pattern reveal the crystalline nature of as-synthesized NPs. The Cr doping slightly alters the intensity of diffraction peaks related to ZnO and reveals a few new peaks of ZnCrO_4_. The estimated crystallite size of the as-synthesized NPs obtained using Debye–Scherer’s formula is 35 nm for ZnO and found between 13 and 22 nm for Cr-doped ZnO. The strain (ε) calculated using Williamson–Hall (W–H) relation (Equation (2)) initially increased for a Cr concentration of 13 × 10^17^ atoms/cm^3^ in comparison to pure ZnO and decreased with the increase in Cr atomic fractions in the ZnO matrix given in Figure 2b.

This indicates that Cr doping initially results in strained crystals owing to a 6.2% lattice mismatch in the crystal and gets compensated at a high density of Cr atoms in the system as evident from the crystal dislocation estimated using the Williamson and Smallman approach, γ = 1/D^2^, and follows the trend as that of the strain (Figure 2b). Table 1 demonstrates the typical crystal and optical characteristics of the synthesized pure and Cr-doped ZnO NPs.

Further, to confirm the functional properties of synthesized material, FT-IR spectra were acquired in ATR mode. A broad peak around 1610–1730 cm^−1^ depicts O-H stretching. The peak at 1420 cm^−1^ suggests C-H bending whereas 800 cm^−1^ indicates C-H vibrations. The metal oxide peak observed at 680 cm^−1^ is due to ZnO stretching (Figure 2c). To monitor any changes in the surface functional properties and conformation of protein, the FTIR study of undoped and Cr-doped ZnO was observed in the presence of 15 nM Mb along with the Mb solution prepared in phosphate buffer solution (PBS) at pH 7.4. This was carried out since conformational changes in the secondary structure result in a change in the surface functional group [50,51]. Figure 2d exhibits typical FTIR spectra of synthesized NPs with Mb. As observed, the pink graph is the spectra of Mb revealing a peak at 1054 cm^−1^ due to C-O stretching; the peak at 1536 cm^−1^ ascertains to N-H stretching which is an amine II band; the peak at 1685 cm^−1^ depicts C=O stretching and the peak appearing below 800 cm^−1^ is associated with heme present in Mb. However, only the peak at 1054 cm^−1^ and 1696 cm^−1^ observed with NPs is shifted towards the high energy side. The significant shift in amide bond energy suggests the formation of a nanoconjugate (Figure 2d).

The optical properties of the synthesized NPs were probed using UV–Vis absorption for the wavelength ranging from 225–450 nm. Pure ZnO reveals a broad absorption band centered at 260.5 nm (black curve) in Figure 2e. The Cr-doped NPs (red, blue, and light purple curves) depict broad symmetric absorption centered at 264.5 nm with trivial red shifts with decreasing doping concentrations in the ZnO matrix. The red shift of 3 nm may be correlated to the agglomeration of NPs as evident from FESEM images (Figure 1). The direct band gap of samples is estimated by plotting (*α*h*ν*)^½^ versus (h*ν*) to demonstrate a decline in the value with doping. A minimum of 2.82 eV is obtained for a Cr doping concentration of 13 × 10^17^ atoms/cm^3^ as observed in Figure 2f. This happened due to crystal stress which induces deep level states and shrinks the band gap of ZnO amid Cr doping. The absorption spectra studied with Mb exhibit an added peak at 409 nm showing that the characteristic absorption of Mb evolves from the heme Soret band [52]. This increased linearly with the Mb concentration as reported earlier by G. Mandal et.al [53].

**Figure 3:**
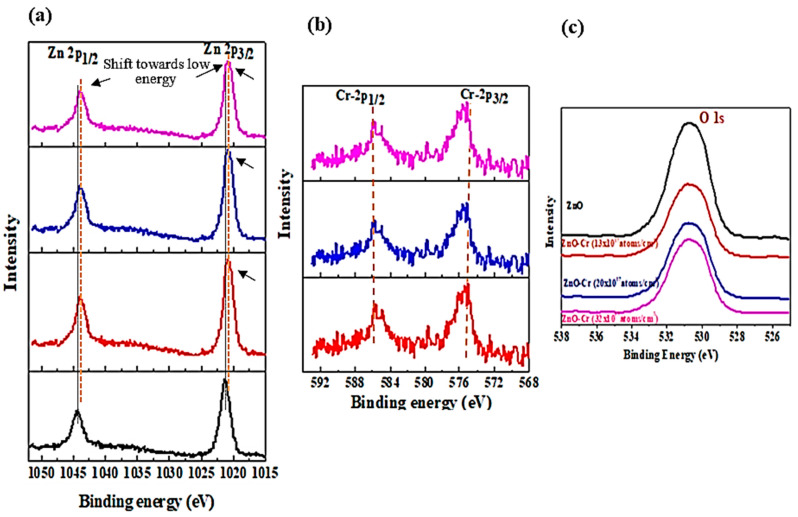
High-resolution X-ray photoelectron spectra for pristine and Cr-doped ZnO (**a**) Zn 2p (**b**) Cr 2p and (**c**) O 1s.

Figure 3 represents the X-ray photoelectron (XPS) spectra of Zn-2p, Cr-2p, and O-1s core-level states of pure and Cr-doped ZnO NPs. Figure 3a reveals the peak of Zn-2p_3/2_ and Zn-2p_1/2_ states at a binding energy (B.E.) of 1021.5 eV and 1044.0 eV, respectively, for pristine ZnO can be correlated with the oxygen-bound Zn ions in the system. The value of the B.E. is on the lower side compared to that of ZnO ions in the bulk system, which is 1022.5 eV and 1045.0 eV, respectively [54]. The peaks of Zn-2p3/2 and Zn-2p1/2 in the Cr-doped sample are shifted towards low energy by about 0.049 eV suggesting oxygen-deficient NPs. A kink in the Zn-2p_3/2_ peak shown by arrows in Cr-doped NPs is linked to the loosely bound Zn ions. Figure 3b depicts the Cr-2p_3/2_ and Cr-2p_1/2_ spectra for doped ZnO NPs. The Cr-2p_3/2_ and Cr-2p_1/2_ peaks appear at a B.E. of 585.78 eV and 575.37 eV, respectively. B.E. values are lower than the corresponding bulk values of 586.9eV and 576.0 eV suggesting that a few Cr ions replace Zn ions as Cr^2+^ in the system of NPs during synthesis [54,55]. The noisy spectra of Cr imply the presence of a low concentration of Cr. Figure 3c is a set of XPS spectra of O-1s for doped and undoped ZnO particles giving a single, almost Gaussian-like symmetric peak at 530.78 eV for pure ZnO that is shifted to 530.68 eV for Cr-doped NPs. This is correlated to the O^2−^ ions bound in the ZnO matrix. The peak shift by about 0.01 eV (Figure 3c) towards low B.E., a noticeable reduction in intensity and peak broadening observed, is ascertained to the loosely bound oxygen ions that might be present in the crystal. The XPS results are inconsistent with the results of zeta potential as the surface charge values increased for Cr-doped NPs indicating the oxygen is adsorbed on the surface.

### 3.2. Fabrication and Characterization of Mb Biosensor Based on Cr-Doped ZnO NPs

**Figure 4:**
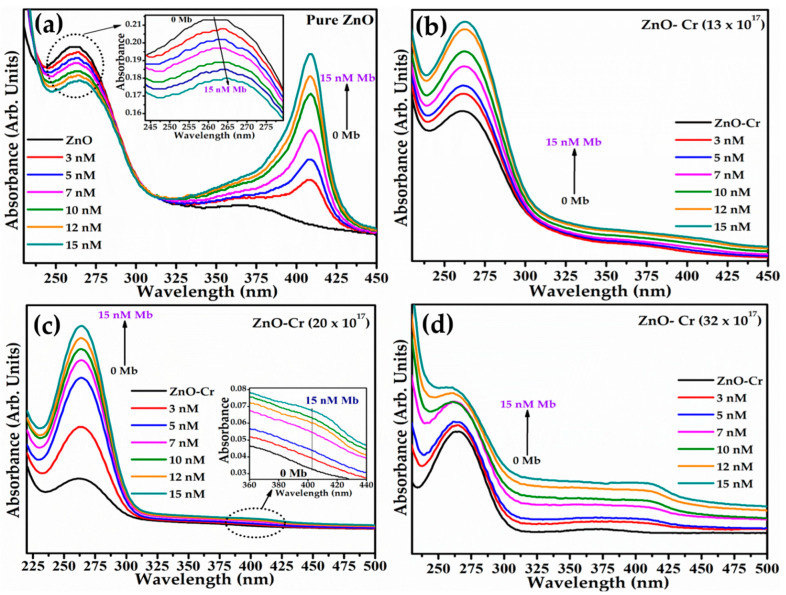
UV–Vis absorption spectra of synthesized pure and Cr-doped ZnO NPs with various concentrations of Mb.(**a**) Pure ZnO, (**b**) ZnO-Cr (Cr = 13 × 10^17^ atoms/cm^3^), (**c**) ZnO-Cr (Cr = 20 × 10^17^ atoms/cm^3^), and (**d**) ZnO-Cr (Cr = 32 × 10^17^ atoms/cm^3^).

The systematized absorption study of nanoparticle titration by the Mb solution was carried out by successively adding 3 nM–15 nM Mb in a 7.4 pH PBS solution of 10 μg/mL ZnO NPs. Figure 4a–d shows the four sets of absorption spectra of doped and pristine ZnO attained at different Mb concentrations. The black spectrum in pristine or Cr-doped ZnO NPs is obtained without Mb (Figure 4a–d). For pristine ZnO, systematic addition of the Mb solution exhibits a noticeable hypochromic shift with a monotonous decrease in ZnO peak intensity as revealed in the inset of Figure 4a. However, in Cr-doped ZnO, a gradual increase in intensity is monitored at a fixed peak position. The increased peak intensity of doped ZnO explains the possibility of Mb–NPs conjugation as noticed from the FT-IR spectra (Figure 2d). The increased absorption at 409 nm in all sets suggests an increase in the density of free/unbounded Mb molecules in the solution with Mb concentration in the solution. The highest intensity of the Mb peak found for pristine ZnO (Figure 4a) is yet to be analyzed.

**Figure 5:**
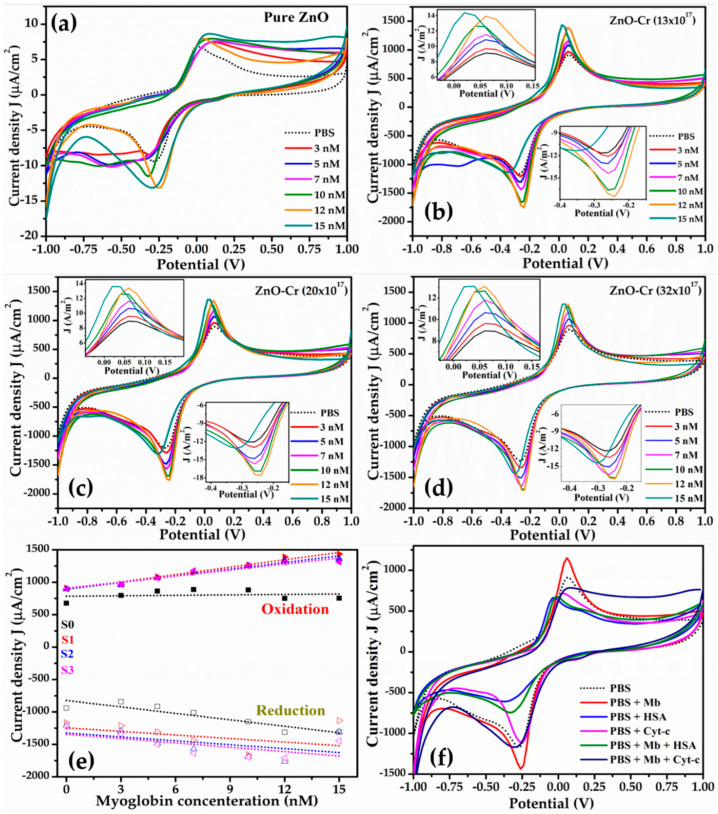
Cyclic voltammetry (CV) curves for pure and Cr-doped ZnO NPs at various concentrations of Mb; (**a**) pure ZnO; (**b**) ZnO-Cr (13 × 10^17^ atoms/cm^3^); (**c**) ZnO-Cr (20 × 10^17^ atoms/cm^3^); and (**d**) ZnO-Cr (32 × 10^17^ atoms/cm^3^). (**e**) Peak oxidation current plotted as a function of Mb concentration (symbol represents experimental data and dotted line is best fit), and (**f**) interference study with HSA and Cyt-c.

To estimate the sensing characteristic, the CV curves for pristine and Cr-doped ZnO were obtained as a function of concentrations of Mb from 3–15 nM and are shown in Figure 5a–d. The dotted black curve represents the electrode response with the PBS buffer demonstrated as a reference. A systematic increase in the magnitude of the oxidation and reduction peak current was noticed with increasing Mb concentration. The inset in Figure 5b–d displays an enlarged view of the oxidation and reduction peaks for doped ZnO. The oxidation potential shows variation from 0.02V to 0.2V and reduction potential from −0.28 V to −0.14 V. For doped ZnO, the peak oxidation and reduction potential is nearly constant as seen in Figure 5b–d. A slight shift in the oxidation and reduction peak potential is observed at a higher concentration of Mb. Moreover, the sensors are highly sensitive at lower concentrations of Mb. The shift in oxidation and reduction potential and increase in peak current at higher Mb concentrations are expected since free/unbound Mb and biomolecules are thin insulators.

The plot of peak oxidation current density (J) against Mb concentration (Figure 5e) shows a linear increase and is used as a calibration curve and sensitivity was estimated by obtaining the slope of the curves. The symbols and dotted line represent the experimental and the best fit data obtained, respectively. Table 2 lists the sensor parameters including sensitivity for electrodes. Doped ZnO by 13 × 10^17^ atomic/cm^3^ exhibits a maximum change in current value correlated as maximum sensitivity for sensing Mb (Figure 5e). The sensitivity is found to be increased upon doping and is found as 37.97 µA-nM^−1^cm^−2^ for 13 × 10^17^ Cr atoms/cm^3^ whereas increasing the doping concentration resulted in decreased sensitivity to 34.31 µA-nM^−1^cm^−2^ for 20 × 10^17^ Cr and 30.89 µA-nM^−1^cm^−2^ for 32 × 10^17^ Cr atoms/cm^3^. The limit of detection is calculated using the slope of peak current versus concentration and the standard deviation, i.e., 3xS.D./slope is listed in Table 2, presenting the lowest value of 0.15 nM obtained for 13 × 10^17^ Cr atoms/cm^3^ with a LOD that is the lowest compared to the reported values so far to the best of our knowledge.

The increase in sensitivity for 13 × 10^17^ Cr atoms/cm^3^ doping concentration and then decrease in sensitivity with a further increase in doping concentration is expected as 13 × 10^17^ Cr atoms/cm^3^ doping exhibits the maximum stress in the system which further reduces with increasing doping (Figure 2b). This intern suggests the presence of maximum density of states for 13 × 10^17^ Cr atoms/cm^3^ doping. The further increase in doping reduced stress in the system by concentration contributes to decreased sensitivity.

Figure 5f shows the characteristic CV curves obtained for the interference study with human serum albumin (HSA) and cytochrome c (Cyt-c) to evaluate the specificity of the proposed sensor. CV curves were obtained for pbs, Mb, HSA, and Cyt-c separately. Further, CV for the mixture of Mb (7nM) with HSA (5mM) and Mb with Cyt in a 1:1 volumetric ratio buffer solution was also recorded. HSA was taken as it is present in abundance in the blood and the reason to choose Cyt-c is because of its resemblance to Mb. The oxidation peak potential of buffer, Mb, HSA, and Cyt-c appeared at 0.06 V, 0.06 V, −0.02 V, and 0.04, respectively, whereas the peak potential value for the mixed solution of Mb with HSA and Cyt-c was −0.02 V and 0.08 V, respectively. The sensor exhibits a distinct response to the HSA and Cyt-c.

**Figure 6:**
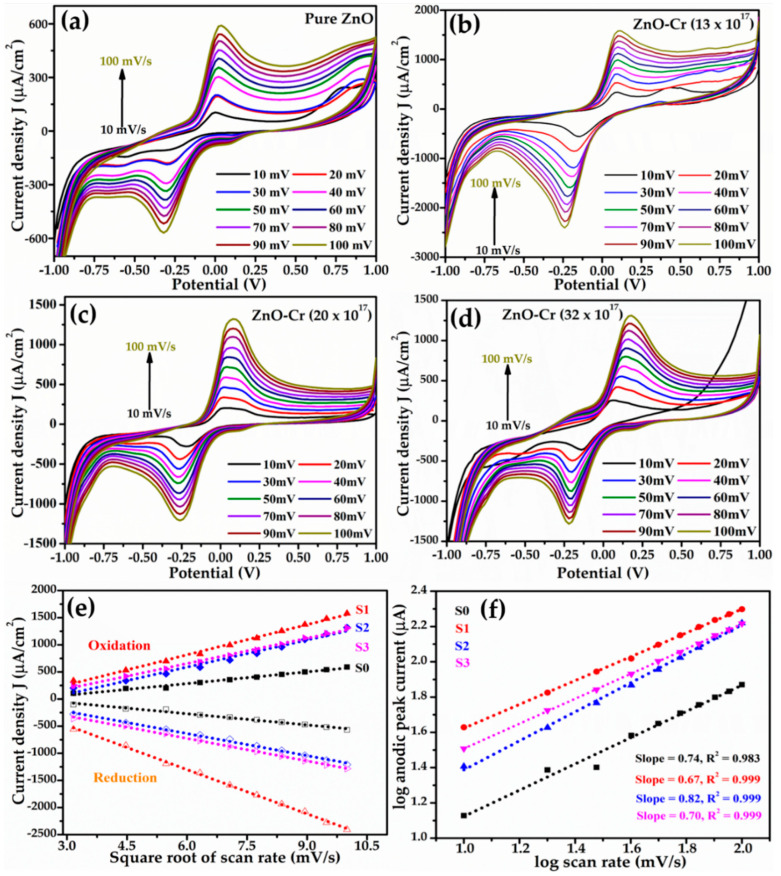
Effect of scan rate on the CV characteristics obtained at 7 nM of Mb for the electrodes made from (**a**) pure ZnO; (**b**) ZnO-Cr (13 × 10^17^ atoms/cm^3^); (**c**) ZnO-Cr (20 × 10^17^ atoms/cm^3^), and (**d**) ZnO-Cr (32 × 10^17^ atoms/cm^3^). (**e**) Peak current (both oxidation and reduction) versus square root of scan rates, and (**f**) log of peak current (oxidation) versus log of scan rate. The symbol represents experimental data points and dotted line is the best fit obtained.

In order to assess the charge-transfer characteristics, CV curves were extorted at 7 nM Mb concentration by varying the scan rate from 10 to 100 mV/s and are depicted in Figure 6a–d. A monotonous increase in the magnitude of peak current for both oxidation and reduction is detected with an increasing scan rate irrespective of the samples. The fast scan rates demonstrate increased current as a result of lowering the thickness of the diffusion layer. The marginal shift of oxidation potential towards higher energy with the increasing scan rate might have several reasons. However, it is correlated to the change in charge-transfer characteristics and hence the change in reaction kinetics to balance the reaction occurring at the electrode surface. The increase in potential is predictable due to the increase in effective thickness by immobilization on the electrode surface during sensing. The change in |E*_a_*-E*_c_*| suggests a change in the kinetics of the reaction; nevertheless, the reaction gradually acquires reversibility (Figure 6a–d). The same trend is obtained for all NPs. The scan rate CVs predicts the electron transfer processes during the reactions that are electro-chemically reversible and involve redox species that freely diffuse and do not deposit over the electrode surface.

The graph of peak oxidation/reduction current density versus square root of scan rate describes the smooth variation over the studied range illustrated by the linear fit (Figure 6e). This implies a typically diffusion-controlled charge-transfer process at the electrode surface [64]. Moreover, a plot of the log of peak current as a function of the log of scan rate (Figure 6f) is found linearly fitted with a slope of 0.56 in the proximity of the theoretical value of 0.50 for the diffusion-controlled process [65]. Nevertheless, a slope of 0.67 or above suggests that the process is not solely diffusion controlled but that adsorption also contributes as some redox species might be adsorbed/deposited on the electrode surface due to the non-uniform distribution of ions associated with the rate-limiting process giving low sensitivity.

The Randles-Sevcik equation given below is used to estimate the diffusion coefficient for pristine and doped ZnO as the CV study predicts a reversible electron transfer process

$$i_{p}=0.4463.F.A.C.n^{3/2}{\left(F\nu D/RT\right)}^{1/2}$$

where *i_p_* is the highest value of current (Amp), *n* is the number of free electrons involved in the reaction, *A* is the electrode area in cm^2^, *F* is Faraday’s constant, *C* is the molar fraction of solution, *D* is the diffusion coefficient (cm^2^/s), *ν* is the scan rate (V/s), *R* is the universal gas constant (JK^−1^Mol^−1^), and *T* is the temperature in °K.

The average value of D for pristine ZnO is 5.5 × 10^−5^ cm^2^/s, for 13 × 10^17^ Cr atoms/cm^3^ in the ZnO matrix its value is 2.19 × 10^−6^ cm^2^/s, for 20 × 10^17^ atoms it is 1.53 × 10^−6^ cm^2^/s, and for 32 × 10^17^ Cr atoms/cm^3^ it is estimated at 1.50 × 10^−6^ cm^2^/s. The increase in the value of the diffusion coefficient determines the concentration variation in the system and hence the apparent electron diffusion kinetics of the chemical reaction with the doping concentration.

**Figure 7:**
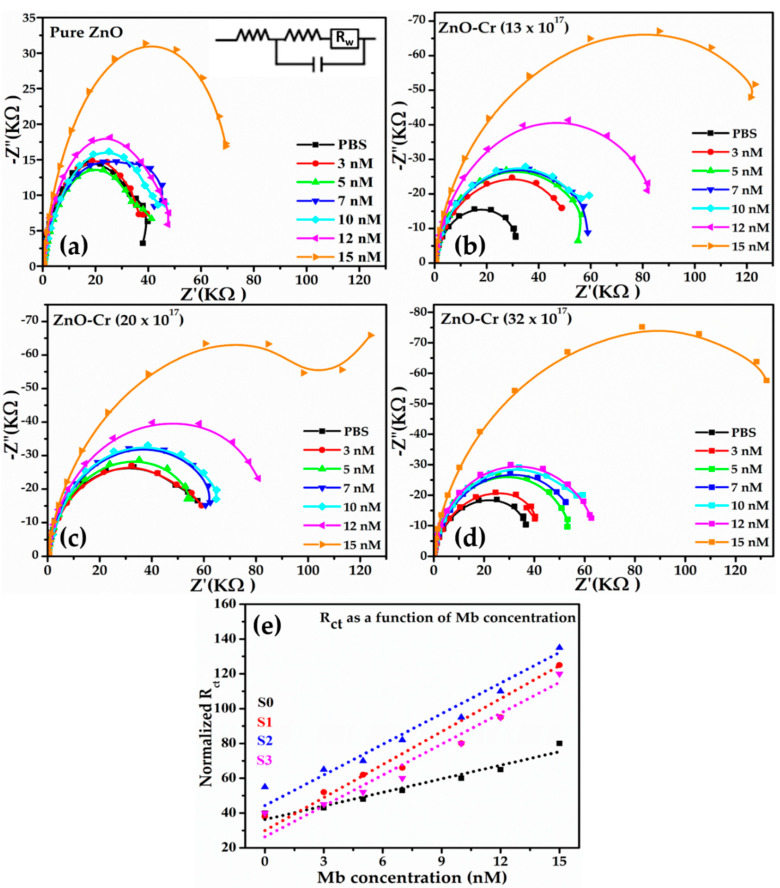
Series of Nyquist plots acquired for different Mb concentrations. (**a**) Pure ZnO; (**b**) ZnO-Cr (13 × 10^17^ atoms/cm^3^); (**c**) ZnO-Cr (20 × 10^17^ atoms/cm^3^), (**d**) ZnO-Cr (32 × 10^17^ atoms/cm^3^), and (**e**) *R_ct_* values for different Mb concentration.

To investigate the effect of doping concentration, an EIS study was separately carried out for all NPs for 3–15 nM Mb concentrations. Figure 7a–d presents the four different sets of EIS data by plotting the imaginary part of impedance Z (Z″(KΩ)) versus the real part of Z ((Z′(KΩ)) (Nyquist plots) for pristine ZnO and doped ZnO.

Typically, for all the samples, the radius of the high-frequency semicircle, known as the charge-transfer resistance (*R_ct_*), at the interface between the electrolyte and sensing layer increases with increasing the Mb concentration and is interrelated to the increase in the charge-transfer density because of the high Mb concentration in solution. This trend is valid for all the samples. The linear region at the low-frequency range ascertains diffusion. For pure ZnO, variation in the *R_ct_* value with Mb is observed low as compared to the Cr-doped ZnO (Figure 7e). The minimum variation of R*_ct_* values observed for pristine ZnO (Figure 7e) implies low charge-transfer characteristics leading to low sensitivity (Figure 7e). The trend of *R_ct_* values was lowest for pristine ZnO and highest for doped with 20 × 10^17^ followed by 13 × 10^17^ and 32 × 10^17^ Cr atoms/cm^3^ in the ZnO matrix.

The trend of sensitivity for samples is in accordance with the maximum value (−20.6 mV) of the zeta potential (Figure 1e, Table 1) and the highest dislocation density (Figure 2b, Table 1) for Cr-doped ZnO with 13 × 10^17^ atoms/cm^3^.

**Figure 8:**
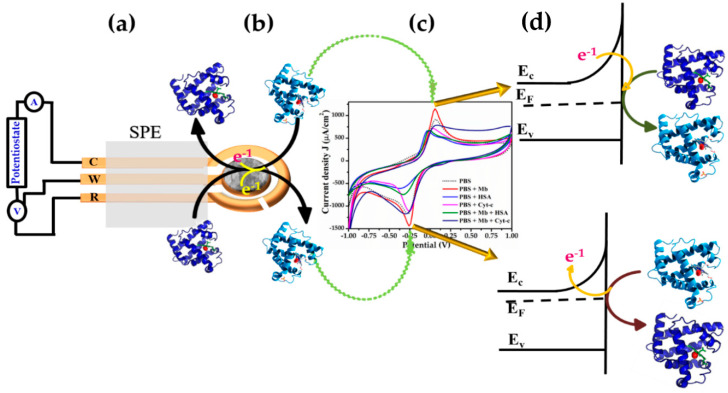
Schematic mechanism for the detection of Mb using pure and Cr-doped ZnO NPs.

Figure 8 is the proposed sensing mechanism illustrating the sensing properties and electron transfer characteristics taking place during the redox reaction. Figure 8a exhibits the typical picture of the modified SPE electrode. In an open atmosphere, Mb is oxidized due to the presence of Fe-ion; a known redox couple. During the CV, when the applied potential at the electrode crosses the E_1*/*2_ value, the electron is transferred from ZnO to Mb translating it from Fe^3+^ to Fe^2+^ reducing Mb and oxidizing ZnO causing increased oxidation current. In this case, the depletion region reduces as ions are diffused from the electrode to the bulk solution. The increase in the degree of oxidation is also elucidated from the absorption obtained during Mb titration where the ZnO peak broadens by the expansion of the electron cloud (Figure 4a–d)**.** In addition, the XPS study revealed the presence of loosely bound electrons in the NPs system.

In contrast, during negative sweep as the applied potential reaches equilibrium, the electron is transferred from Mb to ZnO oxidizing Mb during Fe^3+^ state to Fe^2+^ state by releasing an electron to ZnO resulting in a reduced current (Figure 8b–d). In this case, ions diffuse from bulk towards the electrode surface leading to the growth of the depletion layer hence a reduction in current. As indicated from the Randles-Sevcik equation, the process is based on single-electron transfer and is reversible in nature. The different current values and shift of peak potentials found during the scan rate can be interrelated to various interfering factors that arise from the surface and physical status of electrodes with time.

## 4. Conclusions

In summary, pure and Cr-doped ZnO NPs were synthesized via the facile sol-gel method and used to fabricate high-sensitive and selective acute myocardial infarction myoglobin biomarkers. The synthesized NPs were examined by various techniques which confirmed the spherical-shaped morphologies, well-crystallinity, and high purity. The optical band gaps of the synthesized NPs were calculated using Tauc’s plot which confirmed that the band gap energy decreases upon increasing the Cr-doping concentration. The fabricated Mb biosensors based on Cr-doped ZnO NPs demonstrated around three times more sensitivity than the pure ZnO NPs-based sensor, according to comprehensive electrochemical measurements. The Cr-doped ZnO NPs-based Mb sensor with 13 × 10^17^ Cr atomic/cm^3^ has a maximum sensitivity of ~37.97 μA·cm^−2^nM^−1^, a limit of detection (LOD) of 0.15 nM, and a reaction time of 10 ms. The interference study with Cyt-c and HSA revealed distinct output characteristics than that of Mb which exhibits selectivity towards Mb sensing. The recent work revealed that Cr-doped ZnO NPs may be used as a sensing matrix to detect AMI rapidly.

## Figures and Tables

**Table 1:** Crystal and optical characteristics of the synthesized pure and Cr-doped ZnO NPs.

ZnO with Cr Doping Concentrations (No. of Atoms/cm^3^)	Optical Bandgap (eV)	Grain Size (nm) *	Dislocation Density (×10^18^m^−2^)	Strain (ε)	Zeta Potential (mV)
Pure ZnO (S0)	3.59	35	0.0028	0.035	−68.87 ± 0.05
13 × 10^17^ (S1)	2.82	13	0.0061	0.505	−20.60 ± 0.05
20 × 10^17^ (S2)	2.94	22	0.0020	0.293	−53.40 ± 0.05
32 × 10^17^ (S3)	3.29	14	0.0050	0.504	−54.40 ± 0.05

* Estimated from Debye–Scherrer’s formula D = 0.9λ/(Bcosθ); λ-wavelength of X-ray; B is fullwidth at half of maxima of peak at θ the Bragg angle.

**Table 2:** Reported LOD values based on method of detection and sample/matrix.

Method	Method/Sample Matrix	Amplification Signal	Linear Range/Sensitivity	LOD Value	Ref.
SER spectroscopy	Ag nanostructure modified ITO	SER signal	937 R.U (µg/mL)	10ng/mL (0.52 nM)	[56]
Colorimetric biosensor	DNAzyme-gold NPs	Absorbance	2.5–100 nM	2.5 nM	[57]
Potentiometric	molecular imprinted silica beads	Potential	8.0 × 10^−7^ mol/L	1.3 × 10^−6^ mol/L	[58]
Voltammetric	MIP printed glassy carbon electrode	Current	60.0 nM to 6.0 μM (100 μAmg^−1^/mL)	9.7 nM	[59]
Electrochemical	Ti-NT modified electrodes	Current	18 μA mg^−1^ /ml	50 nM	[53]
SPR	Imprinted [poly(HEMA-MATrp)]	SPR signal	0.1 μg/mL–1.0 μg/mL	87.6 ng/mL (10 nM)	[60]
Electrochemical	Peptide immobilized gold electrode	Current	17.8 to 1780 ngml^−1^ (3 μAng^−1^/mL)	9.8 ng/mL (0.5nM)	[61]
Electrochemical	MIP	Current	1nM–1 μM	0.5 nM 9 ng/mL	[62]
Electrochemical aptasensor	AuNPs/BNNSs	Current	0.1–100 µg/mL (40 μA μg^−1^/mL)	34.6 ng/mL	[63]
**Electrochemical**	**SPE-Cr-doped ZnO NPs**	**Current/** **resistance**	* S0: 2.30 µA cm^−2^/nM * S1: 37.97 µA cm^−2^/nM * S2: 34.31 µA cm^−2^/nM * S3: 30.89 µA cm^−2^/nM	**1.030 nM** **0.150 nM** **0.160 nM** **0.163 nM**	**Current work**

* S0: Pure ZnO, S1: ZnO-Cr (Cr = 13 × 10^17^), S2: ZnO-Cr (Cr = 20 × 10^17^), S3: ZnO-Cr (Cr = 32 × 10^17^).
